# Most Important Factors for Deciding Rehabilitation Provision for Severe Stroke Survivors Post Hospital Discharge: A Study Protocol for a Best–Worst Scaling Experiment

**DOI:** 10.3390/mps4020027

**Published:** 2021-05-06

**Authors:** Sushmita Mohapatra, Kei-Long Cheung, Mickaël Hiligsmann, Nana Anokye

**Affiliations:** 1 Department of Health Sciences, College of Health Medicine and Life Sciences, Brunel University London, London UB8 3PH, UK; keilong.cheung@brunel.ac.uk (K.-L.C.); nana.anokye@brunel.ac.uk (N.A.); 2 Department of Health Services Research, CAPHRI Care and Public Health Research Institute, Maastricht University, 6200 MD Maastricht, The Netherlands; m.hiligsmann@maastrichtuniversity.nl

**Keywords:** decision-making, stroke-rehabilitation, best-worst-scale

## Abstract

Efficient decision-making is crucial to ensure adequate rehabilitation with optimal use of healthcare resources. Establishing the factors associated with making decisions concerning rehabilitation provision is important to guide clinical staff towards person-centred decisions for rehabilitation after severe stroke. In this study we conduct a best–worst scaling (BWS) experiment to identify the most important factors and their relative weight of importance for deciding the type of ongoing rehabilitation services a person with severe stroke might receive post hospital discharge. Fractional, efficient designs are applied regarding the survey design. Key multidisciplinary staff regularly involved in making decisions for rehabilitation in a stroke unit will be recruited to participate in an online BWS survey. Hierarchical Bayes estimation will be used as the main analysis method, with the best–worst count analysis as a secondary analysis. The survey is currently being piloted prior to commencing the process of data collection. Results are expected by the end of September 2021. The research will add to the current literature on clinical decision-making in stroke rehabilitation. Findings will quantify the preferences of factors among key multi-disciplinary clinicians working in stroke units in the UK, involved in decision-making concerning rehabilitation after stroke.

## 1. Introduction

People with severe disabilities account for approximately 50% of all stroke survivors in the UK, even though only 20% have a severe stroke at admission [1,2]. They are likely to have worse prognoses due to slow recovery [3] and poor rehabilitation potential [4]. Only up to 40.7% patients with severe disability (modified Rankin score (mRS) = 4) and 17.5% of patients with very severe disability (mRS = 5) make a functional recovery [5,6]. This takes a considerably longer time (approximately 17 weeks for overall participation in activities of daily living (ADL)), compared to survivors of mild and moderate stroke who can have the best functional improvement in 6 weeks [5]. Hence, they require more resources to get back to the community after acute hospital care, having a significantly large economic impact on healthcare services as compared to mild to moderate stroke patients [4,7].

Therefore, ensuring the rehabilitation provision includes decisions regarding what and how much therapy intervention is required, including what level of care and which services are essential for stroke survivors post hospital discharge. Thus, decision-making requires adequate knowledge of the medical condition, appropriate discharge destinations and careful consideration of the desirable outcomes from rehabilitation, including functional independence and psychosocial well-being [8]. Making an effective decision regarding what rehabilitation care is appropriate and who is or is not referred for ongoing rehabilitation services, based on sound justification, is crucial before discharging stroke survivors into the community. This is important for improving the accuracy of forecasting a patient’s future functioning, planning and ensuring appropriate rehabilitation care after stroke [9,10]. Optimal decisions could also add to preventing time loss in accessing rehabilitation and possibly avoiding sub-maximal functional outcomes including improved ability to perform ADL leading to psychological suffering at the individual level [10].

Little is known about how clinicians make decisions for providing rehabilitation care, especially after stroke, with limited available guidelines. A multitude of factors influence the decisions made for providing rehabilitation care while discharging patients from acute clinical settings [10,11]. Prognostic factors such as patient characteristics, severity of stroke symptoms and dependence before stroke are emphasized in the literature for indicating a stroke survivor’s likely benefits from rehabilitation in the early stage post-stroke [12,13]. A patient’s likely prognosis, goals for rehabilitation [14] and social attributes [15,16,17] are important in deciding rehabilitation needs across the stroke care pathway. Whereas, patient values and preferences, the effect of stroke on the patient’s caregivers and the influence of various external factors have been highlighted when making decisions prior to discharging patients into the community. In addition, individual professional expertise in stroke rehabilitation and multi-professional corroboration of shared decision-making plays a crucial role in such decision-making processes for rehabilitation post discharge [18,19,20,21,22]. Thus, the decisions for rehabilitation of severe stroke patients must not be based on known prognostic indicators or rehabilitation requirements alone [15,22,23].

Although various factors are highlighted in the literature that could affect decisions for further rehabilitation post-discharge from clinical settings, specific guidance is lacking for deciding which rehabilitation services and care pathways are appropriate for individuals after stroke [19,20]. Even though predictive tools are valuable in providing information regarding the average pattern of recovery, there has been very limited evidence of the use of any tools in deciding rehabilitation needs for people after stroke [22,24,25]. In addition, shared decision-making by key professionals specialised in stroke care including medical doctors, nurses, physiotherapists, occupational therapists, speech and language therapists and psychologists is recommended by the National Clinical Guidelines for Stroke (NCGS) [26]. However, the actual process on what factors are considered and how decisions are made regarding rehabilitation provision varies in clinical practices.

A recent study qualitatively explored multiple factors the key multi-disciplinary staff considered as influencing their decision-making process concerning rehabilitation of severe stroke survivors [18,22]. Key professionals recommended by NCGS, who are routinely involved in deciding rehabilitation care for stroke survivors, were interviewed to better understand the system that is currently used to determine post-stroke rehabilitation care. Findings explained the interplay of factors at various stages of decision-making [10,12,22,27,28], indicating that clinicians are mindful of the impacts of all the important factors during a patient’s hospital stay, considering the medical factors in the first instance and the patient and their family’s values and desires more thoroughly for deciding post hospital care. The study identified 23 different factors framed under seven categories (Table 1) [18,21,22]. The findings acknowledged that the clinical team considers a long list of factors already established in the literature as well as the patient’s values in a shared deliberation to decide their likely rehabilitation needs post discharge, making it a cognitively demanding, complex process [18,21,22]. Thus, the findings validated the clinical and non-clinical factors determining post-acute care [29,30], while identifying a stroke survivor’s ability to function and their expression of wants and needs as important constructs in guiding decision-making for discharge [20,21].

Decision-making, especially concerning rehabilitation of severe stroke survivors in clinical practice, is currently guided by the experience of the staff with no specific tools to guide the process. Hence, deciding rehabilitation for patients with high levels of disabilities is subject to several ethical dilemmas and subjective biases including the value of the clinicians about treating severely disabled patients [31,32], overestimating or underestimating the impact of stroke on the patient’s health and their expected quality of life [33], fear of litigation and knowledge of the likely outcomes of various interventions [34], which may all contribute to variation in decision-making. However, 23 factors identified in the previous study [22] are too many to consider when deciding on rehabilitation care, which often requires decisions within a short time-frame in a rapidly changing, dynamic clinical environment. It is still not clear whether clinical teams consider all these factors collectively or if one is given preference over the other and which factors are most important to decide rehabilitation post-discharge. Therefore, there is a need to identify the most important factors from a global and individual perspective to help clinicians in effectively deciding rehabilitation care for individuals with severe stroke. Identifying the most important factors would add to effective decision-making by clinicians. Understanding the relative importance of these 23 factors from the clinicians’ perspective will provide insights on which factors need to be prioritised and considered when making decisions, potentially contributing to future consensus activities to derive a framework to guide decision-makers in stroke units. This will not only draw on the experience and validate the established factors as essential, but will also provide the multi-disciplinary staff with a framework on which to base their clinical decisions while keeping them person-centred.

In order to enhance a patient-centred approach, the preferences of clinicians regarding how they value various factors while designing and evaluating rehabilitation care play an important role [35]. In this regard, studies dedicated to understanding the value of health or health-related services (also referred to as preference studies) can provide relevant information to support decision-makers in prioritising the factors to decide best care. This study aims to identify the most important factors and their relative weight of importance for decision-making with regard to which severe stroke survivors would require ongoing rehabilitation post hospital discharge.

## 2. Methods

The BWS technique is a type of conjoint analysis, which is becoming increasingly popular to elicit preferences in health care [36,37]. In this study, we will conduct a best–worst scaling (BWS) experiment, specifically a BWS object case (case 1), also known as an attribute case, to quantitatively assess the relative importance of 23 factors for decision-making for rehabilitation concerning severe stroke survivors, post hospital discharge, which were identified in the literature. A BWS object case is used because factors have no attribute and level structure [13]. BWS studies include series of questions where participants are asked to make choices (hereafter: choice sets), with a minimum of 3 options, in which a person is asked to indicate the best and the worst options [38]. In this study, participants will be asked to complete a series of choice sets in which they have to choose the most and least important factors (for decision-making) from a list of five factors, derived from a master list of factors. There are multiple tasks containing a set of factors in which the combination and ordering of factors differ. These questions suit the purpose of this study as they are robust for scale-related biases, simplify ranking tasks for participants, and effectively discriminate between the ratings of different factors involved in complex decisions [39,40].

### 2.1. Participants

All key decision-makers from a multi-disciplinary stroke team in the UK, as identified by the Royal College of Physicians guidelines [26], will be invited to participate in the online survey. Participants will be eligible to complete the survey if they confirm they are (a) therapists (i.e., occupational therapists (OTs), physiotherapists, or speech and language therapists (SLT)) or medical professionals (i.e., nursing staff or medical doctors) regularly involved in making decisions in a stroke unit; (b) have a minimum of 6 months’ experience working in a stroke unit and (c) are currently working in a stroke unit in the UK and involved in making decisions for the rehabilitation of severe stroke survivors. Participants will be recruited via online platforms (CAPHR hub) and relevant social media channels like Twitter and LinkedIn targeting forums such as RCP, NHS Frontiers stroke community network, the Chartered Society of Physiotherapists (CSP), the Association of Chartered Physiotherapists in Neurology (ACPIN), the Royal College of OTs and SLTs (RCOT & RCSLT), the Stroke Nurses Forum (SNF), the British Association of Stroke Physicians (BASP) and the Society for Rehabilitation Research (SRR). Additionally, participants will be asked to distribute information about the study to the stroke unit head through social media platforms.

The literature provides no guidance as to the minimal sample size for BWS application. Based on previous object case studies, with sample sizes ranging from 15 to 803 participants [37], this study will aim to recruit approximately 100 respondents with at least 40 respondents with a mixture across various multidisciplinary professionals involved in decision-making for stroke survivors, including medical doctors, nurses and multidisciplinary therapists.

### 2.2. The Best–Worst Scaling Experimental Design

This study involves an online survey designed as a best–worst scaling experiment. Due to the number of factors (i.e., 23 factors), fractional, efficient designs will be applied regarding the survey design [37]. In such a design the selection of scenarios (i.e., combination of factors in the choice sets) is structured to generate the maximum amount of information. Software is needed to design such experiments; Sawtooth Software’s SSI^®^ Web platform will be used to design the BWS survey [37]. Fractional, efficient designs are characterized by (1) orthogonality (factors are shown and paired an approximately equal number of times), (2) minimal overlap (minimising the number of times each factor appears within the same set across the design), (3) positional balance (factors appear approximately an equal number of times in each position), (4) connectivity (factors are directly or indirectly linked) and (5) stability (for each survey, four different versions of the questionnaire are used to increase variation). In order to realise a feasible amount of choice sets, fractional, efficient designs are used which result in four different versions of the survey to be generated for the BWS experiment.

### 2.3. List of Factors for Decision-Making after Stroke

In line with previous studies, the doctoral study [18,19,20,21] indicated that there are 23 factors that may influence the decision-making for rehabilitation and care for people with severe disabilities after stroke. In the doctoral study these were categorised into seven categories: (a) medical factors, (b) severity of impact of stroke, (c) stroke survivor’s potential to progress with rehabilitation, (d) external factors, (e) stroke survivors’ and their families’ wishes and preferences, (f) patients’ safety and (g) patients’ needs. These factors were discussed under three sub themes: patient-related factors, social attributes and external factors (see Table 1 and Appendix A for further description). Additionally, semi-structured interviews were conducted with four national experts (i.e., two medical professionals and two therapists) to further validate this list of factors and provide feedback as to what extent the list (1) is complete, (2) has overlap, and (3) is understandable (appropriate wording). This validation activity led to minor amendments (e.g., wording) resulting into the final master list of 23 factors.

## 3. Questionnaire

An online survey was designed via Qualtrics^®^ consisting of a self-administered questionnaire. The structural basis of the survey includes closed-ended questions with an open-ended question at the end to give further comments. Participants are asked to provide online consent to participate and define their professional role at the beginning of the questionnaire. Demographic and professional characteristics (i.e., gender, age, and years of experience) are also assessed.

An estimated total of 15 choice sets are used for each version of the questionnaire, with each choice set composed of five factors from the master list. The order of the questionnaire versions is randomly allocated to participants. Furthermore, at the end of the survey, participants are asked to rate the difficulty of completing the choice sets based on a Likert scale (1 = very easy, to 7 = very difficult). The BWS survey was piloted among 5 health professionals before finalising and was then distributed via Qualtrics^®^.

## 4. Expected Results

Following ethical approval, semi-structured interviews were conducted with four expert clinicians to validate and verify the adequacy of the list of factors in terms of completeness, whether it has overlap, and comprehensibility. The interviewees indicated that the list of factors was complete and had minimal overlap. All interviewees agreed with the factors and mentioned they understood the factors and their description. There were only a few minor suggestions to improve the comprehensibility. The specific descriptions of the 23 factors were therefore adjusted, which led to the final list of factors. This list was then used to design the BWS survey using the Sawtooth Software’s SSI^®^ Web platform. The online survey was designed via Qualtrics^®^ and consists of a self-administered questionnaire. The survey was piloted prior to the collection of data. The survey is currently online and the process of data collection is underway. Results are expected by the end of September 2021. Findings of the study will be presented in local, national and international platforms and published in peer-reviewed journals. Published results will also be shared though various clinical forums for multidisciplinary staff, such as CAPHR and NIHR-CLHARC.

## 5. Discussion

To our knowledge, this is the first study assessing the relative importance of factors concerning decision-making for rehabilitation concerning severe stroke survivors, post hospital discharge. When designing and evaluating rehabilitation care, it is important to understand the preferences of clinicians on how they value various factors, to be able to optimise patient-centred care [35]. Findings will inform about the preferences of (approximately) 23 factors among key multi-disciplinary clinicians working in stroke units in the UK, involved in decision-making concerning rehabilitation after stroke. The research will add to the current literature on clinical-decision-making in stroke rehabilitation. The findings will inform future research and consensus activities to derive a framework to guide decision-makers in stroke units to prioritise the factors for deciding rehabilitation for severe stroke survivors.

The methodology used has several strengths. BWS, compared to the traditional and more widely applied discrete choice experiment (DCE), presents an alternative preference elicitation method [41] that can resolve some of the limitations of the DCE technique [39] and in particular manage a long list of attributes. BWS is relatively simple to understand, thus supposed to reduce the cognitive burden for participants and facilitates the evaluation of maximum-difference questions. BWS overcomes the traditional ‘pick one’ task used in DCE [41] by eliciting additional information on both the most and least preferred options. Additionally, BWS possesses the ability to embrace a larger set of factors to determine preferences [39]. This allows the opportunity to quantify the relative importance of the lengthy list of factors.

This study has several potential limitations. First of all, there is a potential risk for certain groups of staff to be under- or over-represented in the survey due to actual non-response. Second, as the factors used in the BWS study are tailored to the UK setting, the findings of this study may not be generalizable to other countries. Third, as the number of participants may not be equally distributed among the stakeholder groups, the overall ranking of factors may over-represent specific stakeholders. However, subgroup analyses will outline to what extent there are group differences, and what the rankings within each subgroup would be in that case. Fourth, despite the capacity to quantify the relative importance of factors, the best–worst scaling study will provide limited support regarding the level of consensus among the most important factors. Once the factors have been quantified, further validation studies are needed. A Delphi study may be an avenue of future studies to build evidence for co-creation of the decision-making framework. Fifth, the findings of this study assume a universal approach to decision-making, while in practice decision-making may be contingent on other factors, such as the type and severity of stroke. Hence, one needs to be cautious when generalising findings to all settings, stressing the need for future research and nuanced consensus activities. Sixth, with no guidance on the sample size for BWS studies, we derived the number of participants from the range used in previous studies, ranging from 15 to 803 participants [37]. Although smaller sample sizes are suggested for smaller populations (e.g., medical experts), a potential limitation is that the study will be underpowered, which can lead to inaccurate inferences. Seventh, this study aimed to understand the preferences of the multidisciplinary staff involved in decision-making for rehabilitation after stroke. Yet, in light of shared decision-making, future studies may shed light regarding understanding the preferences for decision-making from the patient and family perspective. Finally, participants may not be familiar with the BWS set-up since BWS represents a relatively novel elicitation technique, potentially leading to non-response bias due to opting out of the study.

## Figures and Tables

**Table 1:** Factors influencing decision-making for rehabilitation concerning stroke survivors.

Patient factors	Medical factors	Stroke characteristics
		Status pre-stroke
		Current medical condition
		Comorbidities
	Stroke severity	Severity of impairment
		Impact on function
		Support needed in the community
	Potential to progress	Support from family
		Participation in Rehabilitation
		Potential to recover
Social attributes	Wishes and preferences	Patient’s capacity and wishes
		Family wishes
	Patient safety	Support available
		Resources available
		Support required
	Patient needs	Care needs
		Therapy needs
		Discharge destination
		Change in needs
External factors	Acute organisational	In-hospital pathways and processes
		Service priorities and targets
	Community	Local resources
		Referrals: processes and timelines

## Data Availability

Data not available yet.

## Appendix A. Description of Factors

**Table A1:** Description of factors.

Factors Influencing Decision-Making for Rehabilitation Concerning Stroke Survivors			Description
Patient factors	Medical factors	Stroke characteristics	This might include details of the current episode of stroke, including, type, site and side of stroke, e.g., MCA stroke, R sided CVA, lacunar, etc.
		Status pre-stroke	This might include pre-stroke functional status (in terms of pre morbid MRS score) and activity level of the patient (e.g., independence in mobility/ADL), any morbidity prior to stroke, e.g., diabetes, heart condition.
		Current medical condition	Medical status after stroke, e.g., consciousness, support for breathing (e.g., on trachea), high blood pressure, nutritional status (e.g., enteral feeding).
		Comorbidities	Additional conditions apart from stroke, e.g., cardiac failure, frailty, etc.
	Stroke severity	Severity of impairment	The extent of brain damage due to the current episode of stroke (motor cognitive, visual, etc.) and initial NIHSS and MRS scores.
		Impact on function	How the stroke has affected the patient’s ability to carry out daily activities, general mobility, communication, swallowing, cognitive and psychological functioning.
		Support needed in the community	Support required in the community for further rehabilitation and/or care of severe stroke e.g., continuing intensive therapy, nursing care, etc.
	Potential to progress	Support from family	Involvement or support that can be provided from family network towards continued rehabilitation.
		Participation in rehabilitation	Patient’s ability to engage in rehabilitation including physical ability to engage, motivation and initiation.
		Potential to recover	Likelihood of the patient to recover from symptoms of stroke based on established clinical prognostic indicators immediately after stroke.
Social attributes	Wishes and preferences	Patient’s capacity and wishes	Patient’s ability to make decisions after stroke for their rehabilitation and their wishes and preferences for rehabilitation post-stroke.
		Family wishes	What the family want (wishes and preferences) for the patient’s rehabilitation, e.g., choice of home vs. care home.
	Patient safety	Support available	Types of physical and social support available to the patient in the community, e.g., family/friend available to continue rehabilitation or care, type of housing, etc.
		Resources available	The services and resources available to the patient in the local community to be safely transferred and managed in the community, e.g., community rehabilitation team, charities and peer support.
		Support required	The support that is required for a patient to be able to be transferred to the community, based on stroke specific, multi-disciplinary assessment.
	Patient needs	Care needs	Additional medical and nursing care needs to be looked after in the community, e.g., PEG care, medication management.
		Therapy goals	On-going stroke-specific, MDT therapy and rehabilitation needs and goals, e.g., intensive therapy, psychology, etc.
		Discharge destination	Where the patient will be transferred for further rehabilitation and care, e.g., care home, community hospital with therapy facility.
		Change in needs	Ongoing changes in needs in patient’s rehabilitation and care needs due to improvement or deterioration.
External factors	Acute organisational	In-hospital pathways and processes	Hospital guidelines and pathways that might influence the process of transferring care. e.g., service level agreement with local authorities/regulations, allocated therapists and social worker for stroke units.
		Service priorities and targets	Acute vs. rehabilitation wards/hospitals and their service criteria, e.g., time frame for patient care, length of stay.
	Community	Local resources	What general resources are available in the community to support the patient in the community e.g., what is commissioned by the CCG for the local area, equipment services, etc. (to sustain in the community).
		Referrals: processes and timelines	What referrals to community services are required and how long will they take, e.g., referral criteria for community therapy teams, length of service provided, waiting list, etc.
